# Nanosensors for diagnosis with optical, electric and mechanical transducers

**DOI:** 10.1039/c8ra10144b

**Published:** 2019-02-27

**Authors:** Anam Munawar, Yori Ong, Romana Schirhagl, Muhammad Ali Tahir, Waheed S. Khan, Sadia Z. Bajwa

**Affiliations:** National Institute for Biotechnology and Genetic Engineering (NIBGE) P. O. Box No. 577, Jhang Road Faisalabad Pakistan anammunwar22@gmail.com waheedskhan@yahoo.com sadya2002pk@yahoo.co.uk; University of Groningen, University Medical Center Groningen, Department of Biomedical Engineering Antonius Deusinglaan 1 9712AW Groningen Netherlands yori.ong@gmail.com romana.schirhagl@gmail.com; Pakistan Institute of Engineering and Applied Sciences Nilore Islamabad Pakistan; Shanghai Key Laboratory of Atmospheric Particle Pollution and Prevention, Department of Environmental Science and Engineering, Fudan University Shanghai 200433 Peoples' Republic of China alitahir7@yahoo.com; Nanobiomaterials Group, Ningbo Institute of Materials Technology and Engineering (NIMTE), Chinese Academy of Sciences Ninbgo City Zhejiang China

## Abstract

Nanosensors with high sensitivity utilize electrical, optical, and acoustic properties to improve the detection limits of analytes. The unique and exceptional properties of nanomaterials (large surface area to volume ratio, composition, charge, reactive sites, physical structure and potential) are exploited for sensing purposes. High-sensitivity in analyte recognition is achieved by preprocessing of samples, signal amplification and by applying different transduction approaches. In this review, types of signals produced and amplified by nanosensors (based on transducers) are presented, to sense exceptionally small concentrations of analytes present in a sample. The use of such nanosensors, sensitivity and selectivity can offer different advantages in biomedical applications like earlier detection of disease, toxins or biological threats and create significant improvements in clinical as well as environmental and industrial outcomes. The emerging discipline of nanotechnology at the boundary of life sciences and chemistry offers a wide range of prospects within a number of fields like fabrication and characterization of nanomaterials, supramolecular chemistry, targeted drug supply and early detection of disease related biomarkers.

## Abstract 2

Nanosensors with high sensitivity utilize electrical, optical, and acoustic properties to improve the detection limits of analytes.

## Graphical abstract

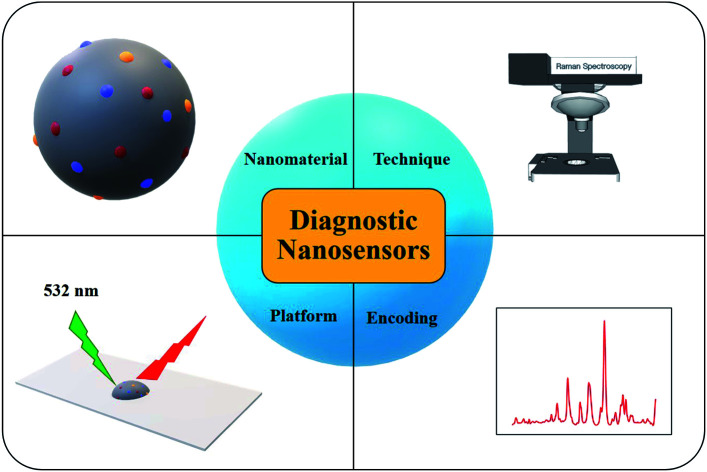

## 1. Introduction

Advances in the era of nanotechnology are moving towards the fabrication of nanosensors that are flexible, specific, versatile and sensitive.^1^ The objective of nanosensors is to screen and measure any chemical, mechanical and physical changes that are related to a marker of interest. Different sensing approaches can be assimilated into other systems like labs-on-a-chip to simplify any kind of detection. The various applications of nanosensors include metabolite monitoring within body fluids,^2^ microorganism detection in different samples,^3^ and finding the pathology of tissues such as tumors.^4^ The ability to detect important molecules, such as disease-related metabolites, proteins, nucleic acids, pathogens, and cells such as circulating tumor cells, is essential not only for disease diagnosis in the clinical setting but also for industrial, environmental and agricultural research development. Nanotechnology, by means of its different properties including increased sensitivity, speed and compact instrumentation size, will promptly expand previous and existing analytical detection range. Nanoscale materials are cost effective, can be selective, and allow multiplexing.^5^ The integration of ultrasensitive nanosensors with other instruments and detection phenomena will increase the competency of emerging nanotechnology to deal with point-of-care type pervasive detection systems.^6^

Along with the different applications there are also different ways to read out nanosensors (*e.g.* optical, electrical, and mechanical) and different ways to manufacture them.^7^ Nanosensing is an interesting and dynamic field to study as the technology is in an early stage, is highly multidisciplinary and has a comprehensive list of applications.^8^

Nanosensors are nanoparticle based devices that sense some kind of signals like force, electrochemical or biological substances. Generally, nanosensors work at nanoscale size. Specificity in nanosensors is imparted by targeting ligands. These ligands are directly conjugated to the nanoparticles. Depending on the functionality of the ligand it attracts a particular marker of interest (analyte), while the nanoparticles contribute the sensitivity, and convert the signals from one form to the other or act as a detector for generated signals.^9^

Traditional diagnostic methods also exist, which are able to provide output efficiency in screening and detection of marker of interest. These traditional techniques are established to measure the biochemical change or recognition based on immunological attraction. Molecular techniques (polymerase chain reaction (PCR) sequencing, cell culture, spectroscopy or blotting) usually demand sufficient reaction time (from few hours to few days), are in some cases difficult to use and might not provide clear and quick results and high stringency required for specific detection of pathogen and related toxin.^10,11^ Microbiological techniques like cell culture and colony counting require ample time compared to other state-of-the-art methodologies, while both approaches have some advantages like accurate and explicit results. Contrarily, improvements in PCR technology, named as real-time-PCR, enable to complete the reaction within a few hours.^12^ ELISA is a well-established approved method due to its sensitivity and selectivity. Despite these properties, it is time consuming (tedious reactions) and costly.^13^ Specificity of different biosensors depends on the presence of some ligand like antibodies or short DNA contrary strand. Many detection technologies also require extensive sample preparation before being able to handle biological samples for example blood, tissues and urine. Additionally, to attain point-of care devices success in developing countries, it is essential that nanosensors are easy to handle in different environmental situations, cheap, sensitive and that they can be used for multiple analytes.^14^

Nanotechnology is able to solve such issues, and already playing a pivotal role for the fabrication of extraordinary nanosensors.^15^

## 2. Nanomaterials

**Fig. 1:**
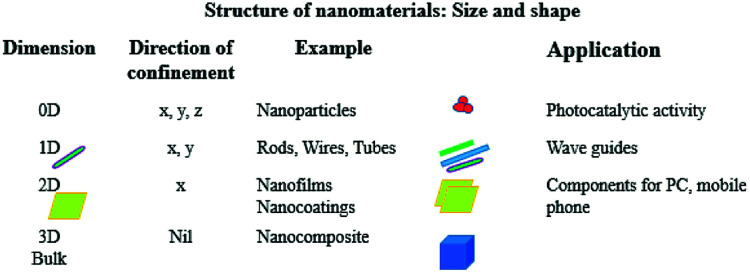
Different shapes of nanomaterials according to their dimensions and their applications.

Now let's focus on some of these nanostructures and their related properties. The following nanostructures are frequently used in the development of nanosensors: nanowires, nanofilms, quantum dots, nanocrystals, nanorods, nanobelts, nanotubes, embedded nanostructures and self-assembled nanomaterials.^16^Fig. 1 gives an idea about different shapes of nanomaterials according to their dimensions and lists a few applications.

This list is not exhaustive, as more nanoparticles exist and new ones are being developed and investigated, but is meant to give an idea about the variety of shapes. The exploration of the new nanostructures with new functionalities is one of the key drivers of nanotechnological developments.

Nanoparticles provide a platform to impart fascinating and unmatched properties in the sensing system such as high reactivity, enhanced electrical conductivity, quantum confinement effects, biocompatibility, different electronic properties, optical effects, strength, exceptional magnetic properties and substantial increase in surface area to volume ratio. Nanoparticles (for instance silica or gold nanoparticles) also offer large relative surface areas that can be functionalised.^17^ Immobilisation on nanoparticles can help to stabilise certain functional molecules. This was demonstrated for instance with enzymes by Liu *et al.*^18^ Immobilising electroactive species in suitable matrixes has been reported to accelerate direct electron-transfer rates.^19,20^ For instance, nanoparticles possess a property to detect high concentration of analyte present in particularly low sample volume due to their high surface area to volume ratio.^21^

2 Dimensional materials are also increasingly important due to their unique ability to conduct electricity and unique quantum confinement effects that come with the 2D structure. Particularly interesting here is graphene and materials that are derived from it.^22^

These new properties and functionalities of nanomaterials allow the fabrication of unique, advanced and astonishing sensing devices and their applications. Furthermore, nanomaterial properties are tuneable by changing their morphology in terms of size, shape as well as chemical and structural functionalities. For example, nanotubes, nanowires, thin platted films, nanorods and nanocantilevers impart versatility, high-sensitivity and selectivity in nanosensors detection systems. Such sensitive detection systems can be used in the area of health research to discover unusual disease related biomarkers.^23^Table 1 gives an overview over different nanomaterials and how they have been utilized for biosensing.

**Table 1:** Different nanomaterials for the detection of different analytes by using various sensing techniques

Target	Nanomaterials	Recognition element	Sensing technique	Ref.
Bacteria	Au NPs	Complementary oligonucleotide	Colorimetry	24
Bacteria	Magnetic NPs	Antibodies	Magnetic susceptibility	25
Microorganisms	Silver nanorods	Electrostatic attraction	Surface-enhanced Raman spectroscopy	26
Toxin	Quantum dots	Single nucleotide chain	Fluorescence resonance energy transfer	27
Spores	Lanthanide doped NPs	Ethylenediaminetetraacetic acid	Photoluminescence	28
DNA	Magnetic NPs	Electrostatic forces	Polymerase chain reaction	29
Pathogenic organisms	Heterogeneous nanowires	Antibodies	Reflectance/PL	30
*M. tuberculosis*	Carbon nanotubes	Complementary oligonucleotide	Impedance	31

Nanotechnology is a field that provide inimitable ways to fabricate sensitive and specific sensing platforms. Nanosensors are also robust and often require smaller volumes than conventional analytical tools. While some of the new nanoplatforms provide unconventional and irreplaceable diagnostic strategies, these systems are mostly not fully optimized for scaling up the fabrication process and commercial applications. In this review, different categories of nanosensors with high sensitivity will be presented. These types are based on optical, electrical, and acoustic signal detection strategies.

## 3. Sensing techniques

Sensors can be classified either based on signal production or by the different methods they employ for signal transduction. Transduction can take place through a number of approaches. There are presently three main transduction approaches categorized based on detection mechanisms: (1) electrochemical detection, (2) optical detection and (3) acoustic/mechanical detection. On the other hand, there is constant progress in designing and optimizing new detection mechanisms of transducers to fabricate new types of sensors. There are different subtypes based on the principle of three main transduction approaches. A number of transduction systems are available in combination with other techniques.^37^ In the following, we give a brief description of the detection systems that are currently available.

A number of studies have been conducted in the field of designing, characterization and optimization of highly sensitive and specific nanosensors for markers of interest. This field opens up a new era of disease prevention and potentially better ways to cure them (Table 2). Limit of detection of nanosensors has extended to pico-(10^−12^), femto-(10^−15^), atto-(10^−18^), and even zepto-(10^−21^) molar scale.^38^

**Table 2:** Nanosensor detection limits for different analytes (SERS = Surface Enhanced Raman Spectroscopy, LSPR = Localized Surface Plasmon Resonance)

Detection method		Nanomaterials	Detection level	Pros	Cons	Ref.
Optical	LSPR	Metal NPs, silica NPs enhanced with Au	pM	Flexible detection	Highly uniform small sized particles (less scattering character)	32
	Colorimetric	AuNPs, AgNPs	nM	Easy to read signal	High probe concentration	33
	Fluorescence	AuNPs-dye, quantum dots	pM	*In vivo* detection	Bleaching or blinking signal	34
	SERS	AuNPs-dye enhanced with Ag, Au–Ag core–shell nanodumbbells	zM	*In vivo* detection	Blinking signal	29
Mechanical		Microcantilever, suspended microchannel resonators	fM	Low sampling volumes	Sensitivity affected by viscous fluid	35
Electrical		Silicon nanowires and nanoribbons, carbon nanotubes graphene sheets	fM	Fast analysis time	Sensitivity affected by salt concentrations	36

**Fig. 2:**
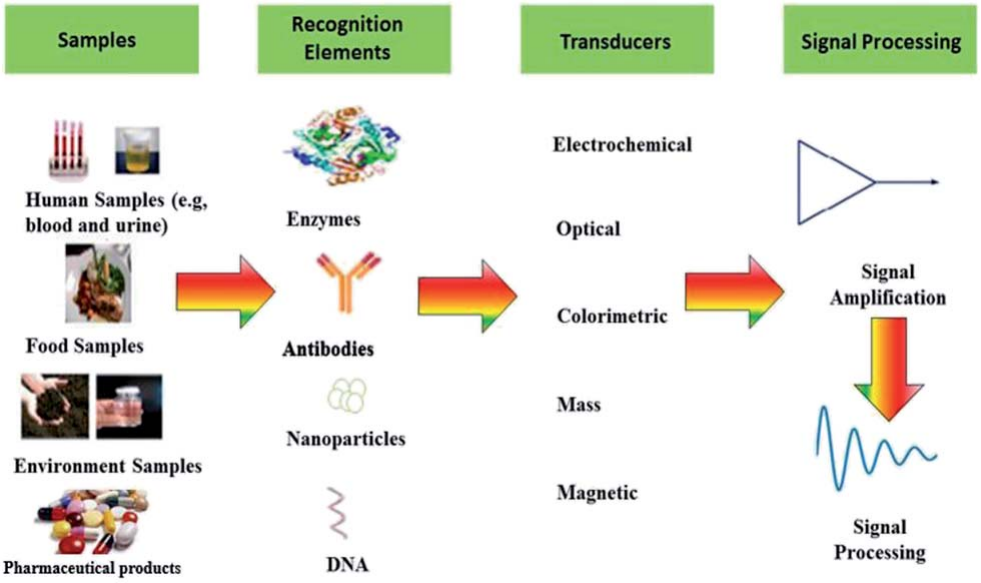
Schematic illustration of the sensing process. Sensors main elements are recognition element and a transduction element. The sensor receives input from the sample, which is converted into a signal. If the recognition element is a nanomaterial (at least one dimension is between 1–100 nm) we obtain a nanosensor.

Table 2 gives an overview over the different transducer principles, different ways of detection and the characteristics of the methods including advantages and disadvantages. Basically, a sensor comprises of two central elements: a recognition element and transduction element. Furthermore, the transducer is linked to a readout system, which transforms or amplifies the measured signal into understandable information for the user.^39^ The general sensing elements described in Fig. 2.

### 3.1. Electrical detection

Electrochemical sensors can be used for numerous analytes. Electrical recognition is a quickly expanding field with built up, basic and minimal effort in the manufacturing procedures.^40^ At present, there are numerous proposed and marketed gadgets in light of the electrochemical approach including those for pathogens,^41^ and toxins.^42^ Electrochemical detection is so popular due to its extraordinary characteristics including sensitivity, low cost, compatibility with modern miniaturized/lab on chip type strategies, least requirement of power, and requiring no-pre sample processing (no effect from turbidity and color of sample).^43^ The standard principle of electrochemical nanosensors is a chemical reaction in which electron release, accept or consume ions. This chemical reaction takes place between a restrained ligand and analyte of interest that measurably affect the transduced signal, such as an electrical current or potential.^44^ This electrochemical signal is directly quantified and related to the presence of marker of interest/analyte in the sample solution. Electrochemical detection approaches have different subtypes on the basis of signal types like potentiometry, voltammetry, amperometry, and electrochemical impedance spectroscopic. Sometimes electrochemical measurements are conjugated with immunoassays. The resulting sensors are called immunosensors. These categories depend on the produced and amplified signal types.^45^

Nanosensors based on detection of electrical signals, first and foremost reported nano field-effect transistors (FETs) possess tunable properties and can be responsible for easy and quantitative measurements. Chemical nanosensors based on FET principle utilize rod shaped nanomaterials (nanowires, nanorods, nanoribbons, nanotowers and nanotubes). When targeted analytes cling to the active area, this results in the change of impedance and produces a signal.^46^ Nanomaterials with these morphologies provide increased sensitivity and active area for current flow, compared to the activity across the cross section of other nano-scale morphologies on the flat detector surface.

**Fig. 3:**
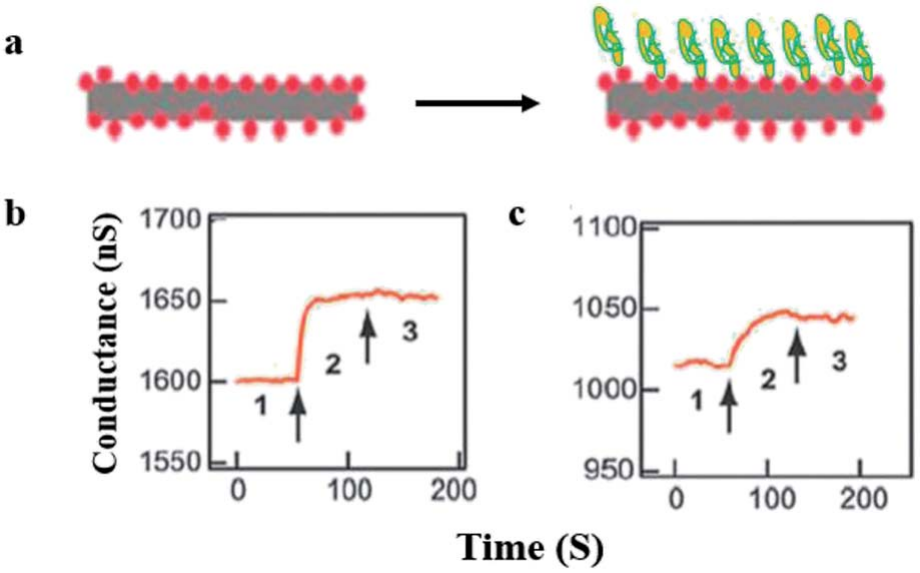
Real-time protein detection by using silicon nanowires (SiNW). (a) Graphic representation of protein attachment (right) to the biotin-functionalized SiNW (left). (b and c) Conductance plotted against time while the buffer solution contains silicon nanowire (region 1) (b) 250 nM to 25 pM of streptavidin protein attracted towards the silicon nanowire (region 2) and as a final point the silicon nanowire releases in buffer solution (region 3). The arrows point toward the changing in solution. (This figure has been reproduced from ref. 48, with permission from AAAS Publishing Group.)

The most commonly used nanomaterial is silicon nanowires because they possess high sensitivity and are easy to functionalize or chemically modify on the surface.^47^ Cui *et al.*^48^ established for the first time the system for direct and sensitive detection in solution by employing nanowires of some semiconducting material (Fig. 3).

This proves that electronic sensors based on nanowire can detect ten pM of macromolecule concentrations, while additional enhancements help to achieve fM limit of detection.

Furthermore, nanosensors can be fabricated and optimized for the detection of proteins on the basis of their conformations. For example conformational changes in calmodulin can be caused by metallic elements. Sensitivity of this kind of sensors relies on the electrical resistance, which originates on the silicon nanowire tips, resulting in a quicker electronic transfer on the silicon nanowire tip as compared to the sidewall. Electrical resistance depends on concentrations ranging from 10 pg L^−1^ to 10 μg L^−1^. The mechanism of action of such sensors is modification in permittivity and electric resistance within the materials on the surface as a result of macromolecule attachment.

Another popular nanosensing material for electrical detection is graphene and its derivatives. Graphene oxide was utilised for the detection of dopamine (a common neurotransmitter which is used as indicator for several diseases, most prominently Parkinson's disease) by Wang *et al.*^49^ In their groundbreaking work they used the π–π interaction between dopamine and graphene oxide to completely eliminate competing ascorbic acid molecules which often pose a problem for dopamine detection. Graphene oxide was also used by Zhang *et al.* in a composite with horseradish peroxidase (HRP) and DNA.^50^ The composite was formed on the surface of a glassy carbon electrode which was used for the detection of H_2_O_2_. In their system HRP reduces H_2_O_2_ while graphene oxide and the DNA stabilises the HRP and facilitates electron transport to the electrode material. In their article the authors were able to detect below 1 mM of H_2_O_2._

**Fig. 4:**
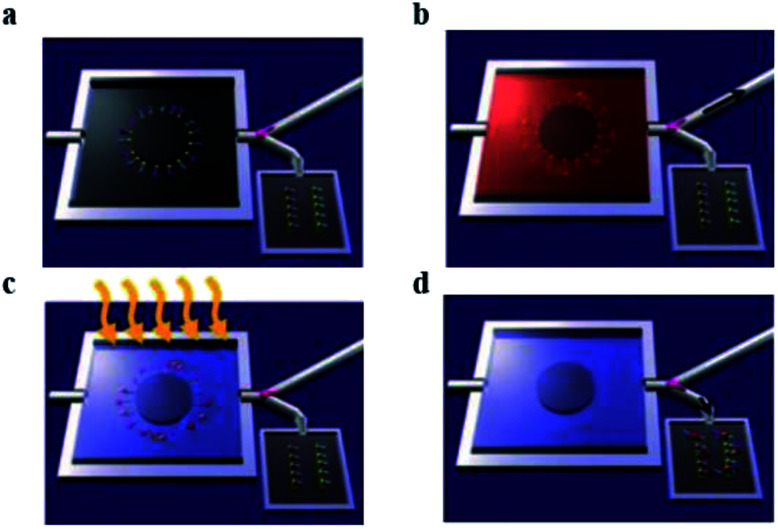
Whole blood analysis for cancer biomarker detection. The device empowers the purification and cancer antigens capture, successive release and transference of the concentrated marker of interest towards the sensing device. Antigens are restrained in the bigger chamber of the microfluidic device. (a) Primary antibodies to multiple biomarkers, are bound with a photocleavable crosslinker. The chip is placed in a plastic housing and a valve (pink) directs fluid flow exiting the chip to either a waste receptacle or the nanosensor chip. (b) The blood sample is introduced in microfluidic device, specific antigens are apprehended by their antibodies. Then washing steps were performed. Antibodies are functionalized by a light sensitive molecule, (c) UV light irradiation used to release these molecules. (d) Conjugates of antigen and antibody are transported towards nanosensors, and an electronic system is used to record the signal. (This figure has been reproduced from ref. 51, with permission from Nature Publishing Group.)

A main limitation in chemical nanosensor handling is that it is not possible to carry out its detection mechanism in physiological solutions, specifically in the presence of high concentration of salts. As an electronic detection system relies on the presence and difference of charge, buffers of different salts will interfere with charge interaction resulted in cut back of nanosensor's sensitivity.^51^ As an example, nanowire FETs need a salt concentration below 1 mM. One approach is to reduce the salt concentration by purifying and pretreatment of sample of interest for better performance of nanosensors.^52^ Stern *et al.* fabricated a microfluidic chip system for purification and to concentrate the analyte. The purified target is then electrically detected. This approach is used for the detection of two different cancer related antigens, 10 μL blood sample was used and results were obtained in less than 20 minutes (Fig. 4).

Such outstanding detection devices work in the presence of complicated physiological conditions. This success is due to the purification microfluidics device that purifies the targeted analyte before scanning by field-effect transistor device. The main function of the chip is to capture the biomarkers of interest from blood or remove other impurities. These purified markers are then released for their electronic sensing. For the release ultraviolet illumination irradiation is used to, break the crosslink between device and analyte of interest by photo cleavage. Sample pre-processing avoids salt interaction with electrical signals, by overcoming this drawback it is possible to utilize cheaper detectors for clinically important biomarkers. However, requiring an extra step clearly will increase analysis time and price.

Although there has been already some progress, further development is needed to handle samples containing high salt concentrations in which sensors should work unperturbed. Physiological conditions can greatly decrease electrical sensing sensitivity. Aggregation of nanomaterials is also a serious issue that needs to be overcome. Contamination is another restriction of carbon nanotubes for electrical sensing. Especially silver from the throughout fabrication process can be problematic. This contamination influences the material quality and disturbs surface modification, increases recovery time, and causes potential irreversible changes to the physical properties caused by surface assimilation. Chemical and electrical nanosensors are proven to be a versatile class of capture assays, these may be transformed in to other forms of nanosensors.

### 3.2. Optical detection

Optical signal detection by nanosensors provides high sensitivity as a result of the distinctive connections of active sites of nanomaterials with light signals. However, sensitivity is strongly depending on the detection mode of the optical phenomena.^53^ Optical sensors are used for several different types of spectrographic analysis, like absorption, visible radiation, fluorescence, Raman, surface enhanced Raman scattering (SERS), refraction, and qualitative analysis using dispersion.

An example of optical signal transduction is the quenching of fluorescence by gold nanoparticles (AuNPs). Fluorescence from fluorescein isothiocyanates (FITC) that move closely to gold nanoparticles is extremely quenched and no visible radiation signal is detected. The same molecule in close proximity to these nanoparticles exhibits increased Raman scattering signals and thus behaves like a Raman probe.^54^ In the following we will introduce highly sensitive nanosensors detection based on optical signals.

Wang *et al.* demonstrated intracellular sensing of adenosine triphosphate (ATP), the molecule that is used as energy unit in the cell.^55,56^ They used a complex of graphene oxide and an aptamer bound to carboxyfluorescein. In presence of ATP the graphene separates from the rest of the complex. As a consequence the carboxyfluorescein is not quenched by the graphene oxide anymore and emits light. The authors were able to follow life cells and detect micromolar levels of ATP.

Surface plasmon resonance (SPR), a common methodology based on analytical chemistry, used to observe molecular interactions forces. It deals with the fluctuations of refractive index due to molecules binding to a thin metal surface.^57^ Light incident on the surface will excite coherent oscillations of surface electrons that are sensitive to electromagnetic fluctuations at the boundary. These fluctuations may be caused by molecule binding events, so that these may give rise to features in the detected reflectivity spectrum. However, it is challenging to attain high throughput sensitivity, because SPR typically has deprived resolution since the unspecified binding interact with the efficacy of material.

An exceptional property of SPR systems unfolds once the light waves intermingle with nanoparticles having smaller size compared to the light wavelength, for example metal nanoparticle.^58^ Possible metals that have been utilised are Au, Ag or Cu.^59,60^ The plasmon system that oscillates around nanoparticles is called localized surface plasmon resonance (LSPR). There are different features of metal nanoparticles (dimensions, morphology and shape), sensitivity of LSPR based sensors relies on.

Small changes that happen due to molecular binding are revealed as a change in excitation spectra of nanomaterials. This distinctive phenomenon can be used for the detection of biomarkers.^61^ LSPR nanosensors are well established and productive multi-array chips (label free detection) and are available for commercial use with 1 nM limit of detection. These illustrations utilize the formation intermingled monolayer of nanomaterial on the substrate. Analytes are captured on the functionalized surface by immobilizing antibodies.^62^ Numerous proteins can be detected through the variation in intensity of absorption LSPR spectra. Examples are immunoglobulins,^63^ C-reactive protein,^64^ and factor I.^65^ Detection is performed when white light shines on the surface of nanochip from a fiber. The reflected light is then collected into the detection fiber, which is coupled into a UV-vis spectrometer for analysis.

LSPR nanosensors require extremely uniform nanomaterials with a narrow LSPR peak to allow for proper calibration. The spectral shift of this peak is characteristic for the analyte that caused it.^66^ As an illustration,^67^ specific silver nanoparticles were used for the detection of amyloid-derived diffusible ligands (ADDL). These are biomarkers for Alzheimer's disease (extracts from human brain) and cancer. The authors used a common principle, a sandwich assay. Such an assay uses a primary protein to capture the analyte and a secondary protein to produce/enhance the signal. Nanosphere lithography was used to synthesize silver nanoparticles with triangular morphology. Mineral substrates provide a platform and functionalization is done by specific protein for ADDL. Once ADDL was captured secondary targeted protein were used to boost LSPR signal. Ultraviolet-visible excitation was used to measure the signals and for qualitative analysis. Optically coupled spectroscope fibers were used for collection and analysis of signals. Silver nanoparticles with specific triangular morphology (having perpendicular cross section of 90 nm and 25 nm height) were selected to increase 35 nm magnetism fields from substrate. It is necessary to notice the distance of captured ADDL within the sandwich assay. This is important since LSPR is strongly depending on the distance. The explained strategy revealed completely different binding coefficients of ADDL protein to secondary protein from brain extracts or tumor. Optical nanosensor detection can be improved by managing the size and form of nanoparticles. LSPR results are helpful for quantitative chemical analysis, distinctive light changes and qualitative analysis for surface-enhanced Raman. The striking advantage of antibodies is that (at least for monoclonal antibodies) every antibody is exactly the same. Thus, unprecedented reproducibility is achieved. The disadvantage is that antibodies are biomolecules and therefore degrade over time and cannot be employed in harsh surroundings. Furthermore, they are relatively expensive and/or time consuming to fabricate.

**Fig. 5:**
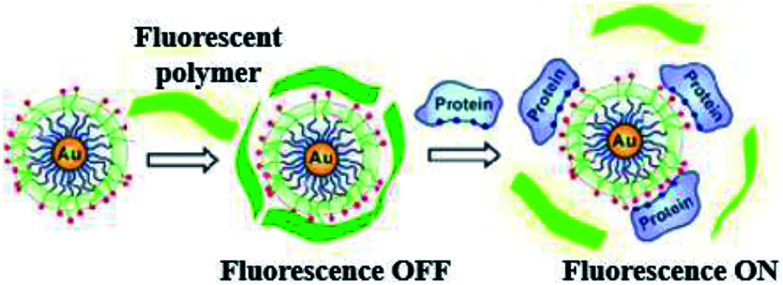
Chemical “nose” sensor. Graphic representation of gold NPs interaction with fluorescent polymer. When the fluorescent polymer intermingles with gold nanoparticles the fluorescence is quenched. When a targeted protein attacks and displaces the polymer, fluorescence is restored. Reprinted with permission from ref. 71, ACS Publishing Group.

LSPR probes use an entirely different optical material property than absorption and scattering.^68^ The latter are employed for detection of markers by using molecular beacons and probes (specific and activatable).^69^ Metal nanoparticles, because of their robust absorption, will quench visible radiation which is produced due to the close vicinity of the surface. Bimetal nanoparticles will increase the visible radiation absorption due to its high scattering cross sectional area. Such kind of mechanisms are quite complicated, different other forms have also been proposed. Metal nanoparticles coated with fluorophores have been targeted to specific sites. These fluorophores are released when nanoparticles surface activated by visible radiation.^70^ A novel technique developed by Rotello and coworkers,^71^ recognized as nanoparticle “noses”, exploits the effects of visible radiation on fluorescent molecules and AuNPs for sensing of different biomolecule like cells, proteins, viruses and bacteria (*in vitro* and *in vivo*).^62,72^ Based on fluorescent probes six AuNP were employed in a fast screening and a differentiation detector array was devised for seven macromolecule targets (Fig. 5).

**Fig. 6:**
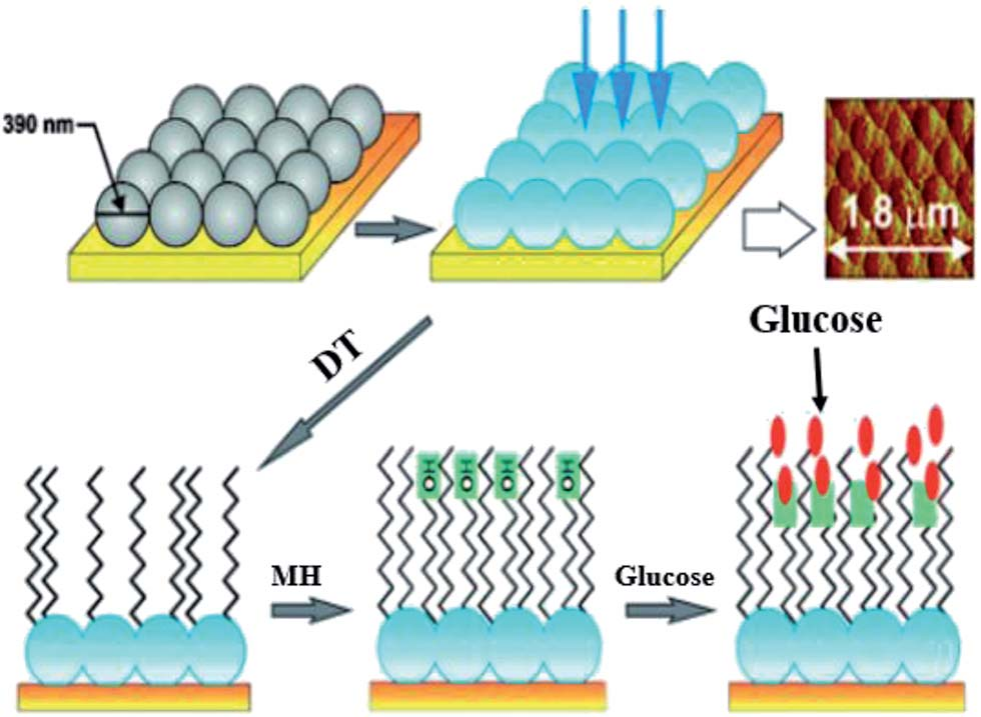
Ag film on nanoparticles sensor: first nanoparticles are coated with silver (blue), then with 1-decanethiol 80 acting as an effective partition layer. The process continues with incubation in (6-mercaptohexane 1-ol) which improves biocompatibility, then the analyte is captured on the surface. Atomic force microscopy is used to show sensor morphology. Reprinted with permission from ref. 80, ACS Publishing Group.

Electrostatic interaction based probes were formed, this interaction is between positively charged AuNPs and negatively charged fluorescent polymers. Six different forms of AuNPs were fabricated with different surface charge due to the functionalization. The specific interactions is due to this difference of charges from the fluorescent compound and consequently selectivity for targeted macromolecules. When fluorescent compounds react with AuNP, visible radiation is quenched. But, due to competitive binding of a target compound, signals can be disrupted and fluorescent compound will be released. These fluorescent patterns are characteristic for particular macromolecules and might be useful for quantification of protein concentrations by a method called linear discriminant analysis (LDA).^47^ LDA is based mostly upon the construct of sorting out a linear combination of the variables that best separates two different categories. There is another class of nanoparticles with some unique properties that are helpful for light and labeling. These are named quantum dots (QDs). These quantum dots are semiconductors, with huge Stokes shifts, wide absorption spectra but nonetheless sharp and broad photoluminescence bands, and high quantum efficiency.^73^ Next generation quantum dots might act as a framework for advanced nanosensors.^74^ However, they are usually to some extent toxic. Rather new alternatives are fluorescent nanodiamonds containing fluorescent defects.^75^ They have two major benefits over the use of fluorescent peroxides or quantum dots. First, emission from FNDs is exceptionally stable; no photobleaching or blinking is observed, even for single defects. Second, diamond nanoparticles are nontoxic to variety of cells.^76^ However, they are usually less bright than standard organic dyes and sometimes are irregular in size and form.^77^ Surface-enhanced Raman scattering (SERS) effect can be observed when a so called Raman signal is boosted by the presence of metal nanoparticle in close proximity. It is possible to detect even the presence of single molecules.^78^ Huge Raman scattering patterns can be produced by using metallic or core/shell nanoparticles with an improvement in the range of 10^14^ to 10^15^. This improvement factor can be attributed to another mode of LSPR, activated at the surface of nanoparticles.^79^ Seo *et al.* modified the SERS field with some advanced features. It can be used for *in vivo* detection of cancer markers. Stuart *et al.* primarily demonstrate *in vivo* detection of aldohexose with SERS, performed in a live rat model (Fig. 6).^80^

Such nanosensors are based on metal-coated nanospheres with a surface functionalized with mercaptohexanol and decanethiol. The surface functionalization leads to preference of aldohexose and reduced unspecific proteins binding. In such types of association the coating thickness is crucial. Detection was performed in the “biological window”, the region of the optical spectrum where biological samples like blood, tissues or cells are transparent. The device based on SERS was inserted subcutaneously for quantifying the aldohexose concentration present in extracellular fluid. The spectra for aldohexose “fingerprint” is improved by applying nanosensors and producing a spectrum outside from the rat's body. Eventually, these devices can provide real time measurements of aldohexose or in the future different forms of metabolic analytes in diabetic patients.

The enhancement in Raman scattering relies on the nanoparticle's spatial separation from the target analyte. Theoretical work shows that the SERS signal is enhanced by stronger electromagnetic fields. This is the case within the proximity of nanoparticles, at opening sites or at the outside of sharp overhangs. Ligands chemically interact with SERS specific active sites, produce a state of charge transfer and an increase in Raman scattering.^28,81^ Doering and Nie found that Cl^−^, Br^−^, and I^−^ ions form a complex with specific nanoparticles and cause SERS spectra enhancement. Citrate, fluoride, and sulfate ions on the other hand cannot produce any enhanced effect on nanoparticles functionalities. Remarkably, a reverse effect was observed by using thiosulfate ions, which resulted in SERS signals quenching. Furthermore, there is an unresolved integral drawback that is related to the stability of nanoparticles. Therefore, a serious hindrance in detection of SERS signals are fluctuations in the frequencies and intensities of signals under different conditions (integration time, particle and analyte concentration, solvent viscosity and density), known as the “blinking” signal.^5^ To solve this issue Lee and coworkers^61^ designed a core–shell material of Au–Ag (nanodumbbells), these nanodumbbells are SERS-active. Deoxyribonucleic acid strands and the location the Raman dye determine the distance between two adjacent particles. The result was consistent, and single molecules can be detected without any hindrance in signals.

Nanoparticles used in optical nanosensors act as a signal production source. Optical nanosensors are thus designed in a way to produce a change on nanoparticle surface, in direct proportion to the analyte concentration. Though, such changes often take place on the surface of extremely uniform nanoparticles. This targeted property has not been completely attained because most nanoparticles are stable only after applying stabilizing agents on their surface.^82^ Due to particle stability issues, detection in presence of physiological conditions (high salt concentrations) can be an issue for optical detection too. High salt concentrations create harsh conditions for many nanoparticles. Though surface chemistry remains a challenge, nanosensors are ready to bypass these disadvantages.^83^ A possible solution could be taking combinatorial approaches (both binding and stabilizing building blocks). Nanosensor signals can be extremely amplified by coupling to nanoparticles having distinctive optical properties. Such sensing approaches are for example useful for early diagnosis of diseases.^84^

A current limitation of optical sensing techniques for uses *in vivo* is the strong background fluorescence from tissues in this wavelength area. A potential solution around this problem is offered by upconverting nanoparticles.^85^ Such particles can be excited in a low energy range but after absorbing multiple photons emit a signal in the visible range. The advantage is that photons in the near infrared (where tissues are transparent) are used for excitation while visible light is detected. This way deep tissue penetration is achieved while sensitive detection in the visible range is possible. For such particles it is crucial, that the upconversion is efficient.^86^ For this to be the case the material should be low in lattice phonon energies, high in chemical stability, and there should be low symmetry of the lattice. Particularly suited materials are small salt crystals (often fluorides, chlorides or bromides), which are doped with rare earth atoms such as Er^3+^, Tm^3+^, and Ho^3+^ ions.^87^ The anion typically should be similar in size to the incorporated rare earth ions to allow efficient inclusion. A further advantage of these upconverting nanoparticles is that they can be combined with contrast agents for other imaging modalities (as for example MRI contrast agents) and thus can be visualised with different methods.^88^ The biggest bottleneck for upconversion nanoparticles is at the moment their rather complex composition, which renders them expensive or difficult to get. Although, so far safety and toxicity has been evaluated very positively, there is still a lack of data especially in the field of *in vivo* testing. Controlling size and shape and thus achieving reproducibility has also been identified to be a major issue.

### 3.3. Mechanical/acoustic detection

**Fig. 7:**
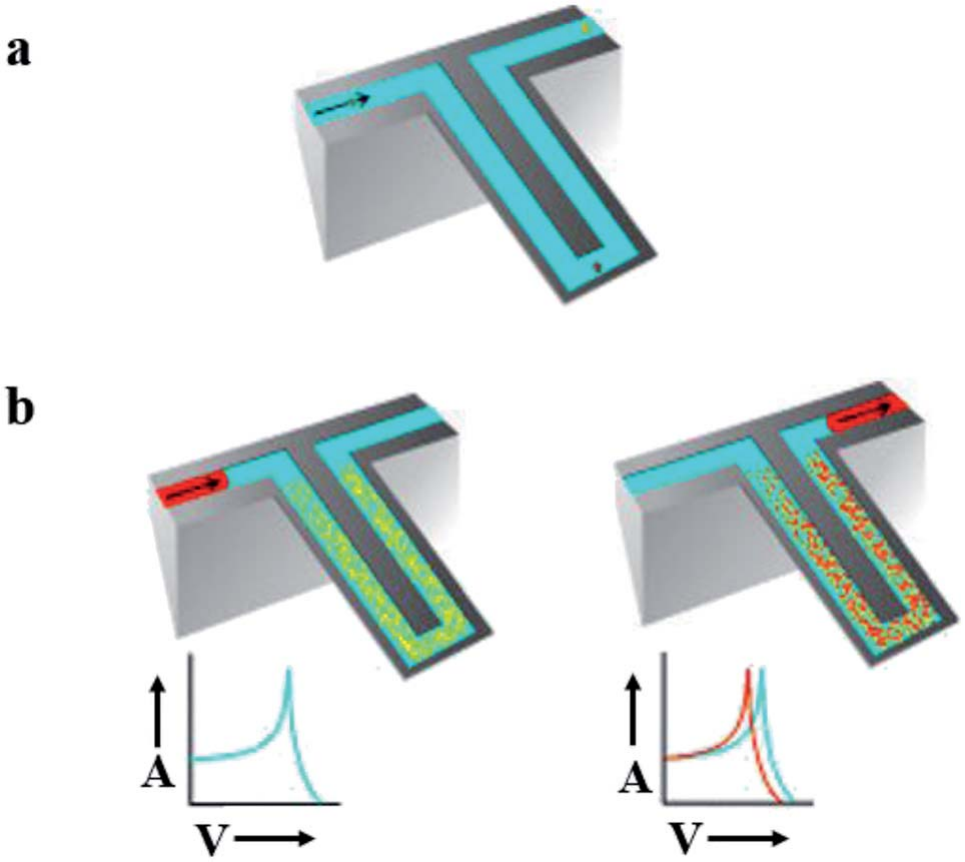
Suspended micro-array resonator. (a) Graphical depiction of suspended microchannel. These channels provide a continuous flow of fluid. Under high vacuum accumulation of mass on cantilever can be detected down to subfemto gram. These mechanical nanosensors solve the issue of detection in fluid. (b) Sandwich assay, targeted molecule accumulate in microchannel resulted in increase of mass (right) while non-targeted molecules continue their flow without any disturbance (left) due to increase in mass frequency shifts. Adapted with permission from ref. 90, Nature Publishing Group.

Mechanical detection systems based on nanoparticles allow ultrasensitive detection and measure the changes in mechanical forces at the molecular level.^4^ Nanomechanical sensors detect forces, displacements and mass changes. The main advantage is that these sensors are sensitive to mass. Their property to measure the mass render them versatile since nearly anything has a mass. Determination of mass by mechanical devices is directly related to overall device mass. Therefore mass detection greatly increases when the mass of mechanical sensors decreases to the nanoscale.^89^ However, detection in fluid faces a major obstacle for mechanical nanosensors. As a result of viscous damping, sensitivity of sensors is critically reduced.^90^ Enclosing fluid filled channels into a cantilever is one option to measure in fluids (Fig. 7).

However, sensitivities of these cantilevers are much lower than what can be achieved in the gas phase. Braun *et al.* developed a sensing array of resonant microcantilevers. This resonance based sensors can maintain their functionality in different physiological condition of liquids. They were used to detect the force of attraction between receptors on transmembrane molecule and respective ligands. This resonance based array sensor was employed against supermolecule receptor on the surface of *E. coli*. Liposomes are the crystallized form of supermolecule receptor of *E. coli*, referred to as proteoliposomes. Ink-jet spotting was used to immobilize proteoliposomes on an Au-coated surface. T5 virus mass can be measured down to pM concentrations. The sensing element was capable to measure the binding of virus with its transmembrane receptor. The microcantilevers provide a micro-array resonance format for fast, specific and sensitive detection. Sensitivity of detection system is enhanced due to the micro-array style. Under physiological conditions (interfering agents or temperature fluctuation), parallel sensing experiments reduce false signals. Thus, this technique became a universal approach for receptor–ligand attraction in the presence of a target molecule proteoliposomes.

For clinical use of mechanical nanosensors, detection approach should be straightforward, cheap, sensitive, specific and fast.^91^ Fluid handling in nanomechanical sensors is limited. To overcome this, supportive polymer coating is used on the silicon resonator and then the actual functionalization of surface is done by injecting an antibody. The resonator is functionalized with immobilized antibodies and the analyte of interest is captured by these antibodies. Due to the adsorption of analyte/marker of interest within the micro-array resonator, the volume of sample solution that is needed is reduced. (Kalpakjian and Schmid, 2014). It is also shown that change in the resonance frequency of microcantilevers is directly proportional to the mass added or biomolecules bound. This microcantilever system was used for the detection of activated leukocyte cell adhesion molecules (ALCAM) in unpurified serum at pM concentrations within one minute.

Mechanical nanosensors' sensitivity and specificity are dependent on the designing and fabrication of constant cantilevers and coating of surface by efficient material for selective target binding, respectively.^4^

The Quartz Crystal Microbalance (QCM) is the most common example of an acoustic device. Since 1959 QCM has been used for different applications in the sensing field. Sauerbrey studied the connection between variations in resonance frequency due to the deposited mass density on sensor surface. The typical parts of classical QCM sensors are thin quartz crystal having AT-cut, an oscillator circuit, electrodes (circular) positioned on quartz disc. Quartz material possesses piezoelectric properties, which deal with the conversion of oscillating electrical energy between circular electrodes into mechanical oscillations of the material.^92^ Due to the high quality of quartz material, mechanical oscillations are usually very constant. Oscillation frequency changes are proportional to the amount of adsorbed or placed mass onto the surface of the gold electrodes on the quartz crystal. These mass based devices are called microbalances due to their high sensitivity. They can measure changes in mass even in the nanogram range. To achieve selectivity one can apply a coating of a material, which is selective for the targeted analyte.^93,94^ Different coating materials, can be used to control the selectivity of QCM. Such an approach makes this type of sensors exceptionally versatile. Even with the widespread applications of QCM, there are some drawbacks that need to be overcome. For example sensitivity enhancement and measuring minor frequency shifts to improve the limit of detection (LOD), is still unsolved. Recently, a fundamental frequency (170 MHz) based on electrode less QCM sensor has been reported having a limit of detection of 67 Hz cm^−2^ ng^−1^.^95^ This electrode less classical QCM sensor is a promising technique.

However, the sensitivity to mass is not just the biggest advantage but also the biggest issue with this technology. Since virtually anything has a mass, a large variety of substances can at least theoretically cause a signal and thus compromise the selectivity. Similar to electrical sensors, high salt concentrations can also here disturb the measurement. Changes in viscosity or attachment of analytes that are not perfectly rigid can cause artefacts and lead to a so-called non Sauerbrey behaviour. To minimize these effects, using controls to eliminate non-specific effects is essential. Furthermore, to improve the efficiency of mechanical sensors, sensitive instrumentation is required to diminish backgrounds noise which can be expensive. Biomarker detection can be hampered due to intrinsic environmental conditions as sensitivity is reduced in detection in fluid compared to detection in vacuum.^96^

## 4. Conclusions

Highly sensitive nanosensors provide unique signal detection and amplification strategies to push the limits of detection to zM concentrations. Such sensing capabilities can be extremely useful to detect biomarkers and to diagnose diseases early on or reoccurrence after a treatment. Examples for nanosensor use include the detection of DNA damage,^97^ cancer,^98^ virus infections,^99,100^ cardiovascular diseases^101^ or Alzheimer disease.^102^ However, in many cases the usefulness of nanosensors has yet to be proven in a clinical setting or even in clinically relevant samples. For electrical detection, high salt concentrations are usually interfering with the measurement. But due to aggregation of nanomaterials the presence of slats is also an issue for other transducer principles. Purity of the nanomaterial can also cause issues. Mechanical sensors would theoretically be universally useful since nearly everything has a mass. However, for mechanical detection even handling liquids poses a problem. Although there are some strategies to overcome this issue the sensing performance in liquid is still not as great as in vacuum. Mass production is greatly limited because little to no nanosensors have been scaled up and manufacturing is costly. Additional functionalities can be assigned to nanosensors to go beyond diagnostic applications and towards therapeutic agents leading to so-called theranostics.^103,104^ Before they can be used in medical applications stringent toxicity studies are also required to address the full cycle a nanoparticle takes *in vivo* from uptake and metabolism to clearance.

## Conflicts of interest

There are no conflicts to declare.

## Supplementary Material
